# On Hepatic Fistulæ

**Published:** 1871-05

**Authors:** M. Signerolles


*On Hepatic Fistulæ.* By M. SIGNEROLLES.

(Archives Generales de Medicine, Juin.)

---

Umbilical fistulæ are rare; they may follow an inflammation of the abdominal wall, a fecal abscess, permeability of the urachus, &c. There is also biliary fistulæ, which is still more rare. We are therefore induced to publish a case reported by M. Signerolles in his recent thesis. The author, after having discussed fistulæ succeeding a calculous swelling, an hyatid cyst, an abscess of the liver, reports the following case:

"The subject of this report was a woman of a lymphatico-sanguine temperament, who had complained of pains in the abdomen for seven or eight months. About three weeks before her admission under the notice of Dr. Signerolles, she observed a small swelling below the umbilicus. The woman had never suffered from hepatic colic or jaundice; the pains of which she complained not being generally accompanied by alternating diarrhœa and constipation.

"At her admission, towards the end of December, the patient presented a tumor situated in the umbilical region, of the size of an adult's fist; this diminished slightly under the influence of baths, poultices, and rest. At first very red and painful on pressure, it soon lost its inflammatory character. After an interval of nine days, this tumor commenced to increase in size again; the skin became soft and thin; and the tumor, after opening spontaneously, gave exit to pus mixed with blood and solid fragments (albumen and fibrine). Another fistulous opening was established a few days after the first, and the two orifices, which were situated, the one about two fingers' breadth, and the other about two centimetres, from the umbilicus, were some centimetres apart. A probe introduced into one of these openings could be passed to a depth of seven centimetres, and then arrived at a hard, bony wall. The probes introduced at the same time by the two openings could be brought together at the bottom of the cavity.

"The general condition of the woman was satisfactory; she was, however, pale and somewhat jaundiced, and was also slightly emaciated.

"In the presence of these symptoms, Dr. Richet, under whose care the woman was, at once took into consideration the diagnosis of acute phlegmon, a glandular abscess, a chronic abscess, an acute cancer, a syphilitic tumor, a fecal tumor, or fœtal cyst.

"He rejected at once the idea of acute phlegmon, on account of the local inflammatory symptoms and the slow course of the tumor.

"A glandular abscess commences in induration, followed sooner or later, by suppuration.

"Acute cancer occurs most frequently in the female mamma. It gives off particles of cancerous tissue, which, however, was not the case in the subject of this report.

"A syphilitic tumor is generally as slowly developed as was this subumbilical tumor, but is accompanied by a coppery tint of the skin, is less firm, and always preceded by symptoms of syphilis, which, in this case, could not be made out.

"The diagnosis as to a fecal tumor was more difficult. There was, indeed, a probability that this woman had an intestinal affection. There might have been ulceration of a portion of intestine that had contracted adhesions with the abdominal wall and determined a fecal abscess. But the pus discharge from the tumor of this patient had no particular smell; it was not mixed with fecal matters, and these could not, in the case of fecal abscess, explain that hard portion against which the probe struck, except by admitting the presence of several small pieces of bone in the abscess.

"The existence of a fœtal cyst was a more probable diagnosis, to which M. Richet held.

"As the tumor continued to discharge a large quantity of pus, M. Richet united the two fistulous openings by making a long incision; the finger, when introduced into the wound, penetrated into a track directed upwards, and to the right side, at the bottom of which could be felt a free, hard body with an unctuous feel. This body was movable, and presented angles which were buried in the granulations. M. Richet attempted to extract this with forceps, but did not succeed; the instrument glided over a part of the body without seizing it. This exploration gave rise to an abundant flow of blood.

"On the following day the body had approached nearer to the cutaneous orifice, so that it could be readily felt with the finger.

"Six days later the patient presented to Dr. Richet, at his visit, a hard black body of the size of a die. This was at once recognized as a biliary calculus. Dr. Richet sought both with his finger and probe whether there were not other bodies of a like kind, and then felt a second calculus. Some days afterwards this was discharged from the external opening. M. Richet subsequently removed with forceps a third calculus resembling the former two. The wound, when explored, presented no hard or resistant body, and nothing could be felt by the finger, save granulations.

"The wound soon closed, and the patient, about two months after her admission, was discharged as completely cured.

"It should be noted that the expulsion of the calculi caused no pain; that the patient had never marked jaundice, and that at no period of the illness did the feces present any special characters."

With regard to hepatic fistulæ, it may be remarked that the external orifice is sometimes found at a great distance from the hepatic organ. M. Kamarion, of Strasburg, has collected 35 reports of thoracic fistulæ having their origin in the liver; of these, 23 were due to abscess of the liver, 12 to hydatid cysts.

The abscess of the liver discharged itself in 8 cases into the pleura, twice into the pericardium, and always caused death. Out of 12 cases of the passage of pus into the lungs, there were 6 recoveries.

Out of 12 cases of hydatid cyst discharging itself into the chest, 10 burst into the right lung, 1 into the pleura, and 1 into both pleura and lung. In 3 out of 10 cases, in which hydatids were expelled by the bronchi, was this discharging followed by recovery.

